# Non-traumatic atlantoaxial dislocation co-existing with hypoplastic and high riding vertebral arteries: a case report and review of literature

**DOI:** 10.1097/RC9.0000000000000210

**Published:** 2026-02-19

**Authors:** Hoang Gia Du, Nguyen Van Trung, Nguyen Duc Hoang, Vu Xuan Phuoc, Le Dang Tan, Trinh Minh Duc

**Affiliations:** a Vinmec Smart City International Hospital, Hanoi, Vietnam; b Orthopedic and Spine Surgery Department, Bach Mai Hospital, Hanoi, Vietnam

**Keywords:** atlantoaxial dislocation, case report, cervical fusion, high-riding vertebral artery, vertebral artery hypoplasia

## Abstract

**Introduction:**

Non-traumatic atlantoaxial dislocation (AAD) is an uncommon condition characterized by abnormal displacement between the C1 and C2 vertebrae, resulting in upper cervical spine instability with the possibility of causing mortal injury.

**Case presentation:**

We report a 63-year-old woman presented with chronic mechanical occipitocervical pain and mild upper-extremity weakness lasting more than 20 years. Her vital signs and systematic examination were unremarkable. Neurological examination revealed mild weakness of upper-extremity weakness (grade 4/5), hyperreflexia, impaired fine motor control, hand numbness, and a positive dynamic Hoffmann sign. Preoperative CT-angiography demonstrated left vertebral artery hypoplasia and right high-riding vertebral artery associated with atlantoaxial dislocation. The patient underwent C1–C2 fusion using a hybrid construct: a C1 lateral mass screw and C2 translaminar screw on the HRVA side, and a transarticular screw on the contralateral side. Intraoperative neuromonitoring was unavailable; therefore, the procedure was guided carefully using real-time C-arm fluoroscopy. Postoperatively, her neurological symptoms resolved completely.

**Clinical discussion:**

Anatomical variants of vertebral artery significantly increase the risk of iatrogenic vascular injury during atlantoaxial instrumentation. Surgical intervention in this setting requires adequate meticulous preoperative imaging assessment and surgical planning as well as experienced surgeons to achieve a safe and successful fixation.

**Conclusion:**

Atlantoaxial fixation in non-traumatic atlantoaxial dislocation with high-riding and hypoplastic vertebral artery carries a high risk of vascular injury. Comprehensive vascular assessment and individualized planning are essential to achieving safe instrumentation.

## Introduction

Atlantoaxial dislocation (AAD) is an unusual condition with the abnormal displacement of C1 and C2 vertebrae; and it can cause neck pain and spinal cord compression, even irreversible neurological deficits. It is usually caused by traumatic occurrences or several pathologic diseases unrelated to trauma, especially in rheumatic diseases^[^1^]^. Non-traumatic atlantoaxial dislocation (NTAAD) could result from rheumatologic inflammatory, post-infectious, neoplastic, or genetic diseases^[^2,3^]^. Regardless of the underlying cause leading to AAD, surgery remains an essential treatment of choice. Due to the sophisticated structure of the C1–C2 complex, the surgical approach of this region is a challenge. In the case of vertebral artery variants such as high-riding vertebral artery (HRVA) or vertebral artery hypoplasia (VAH), the surgical technique required is more complicated^[^4^]^. To our knowledge, there are several cases reported of AAD with the anomalous vertebral artery (VA) treated by surgery^[^4,5^]^; however, there is no report of patients with neurological compromises due to AAD with HRVA and VAH simultaneously in the literature.

**HIGHLIGHTS**

- Atlantoaxial dislocation associated with vertebral artery anomalies poses unique surgical challenges.
- Meticulous surgical technique and real-time intraoperative fluoroscopic guidance are crucial when intraoperative neuromonitoring is unavailable.
- Perioperative CT-angiography accurately delineates vertebral artery anatomy and helps minimize the risk of iatrogenic injury.
- Individualized surgical planning are essential to minimize complications.

The uniqueness of our case lies in the occurrence of two types of vertebral artery variants in a patient with NTAAD, as revealed by multi-slice computer tomography preoperatively. This finding highlights the potential lethality of injuring the sole VA during the C1–C2 screw fixation procedure^[^6^]^. This case was successfully managed using C1–C2 trans-articular screw fixation on the VAH side combined with C1 lateral mass screw and the C2 translaminar screw (Wright’s technique) on the contralateral side. Additionally, we provide a literature review, focusing on the management strategy and surgical technique. The work has been reported in line with the SCARE criteria^[^7^]^.

## Case presentation

A 63-year-old woman was admitted to our hospital with a history of progressive, refractory of neck pain and numbness of bilateral hands persisting for a year. She has no history of neck trauma or rheumatic diseases recorded. The patient had suffered from intermittent neck pain radiating to the occipital region for many years prior to the onset of numbness in both hands and seeking medical support. Additionally, she experienced progressive mild constipation and urinary retention over the past several months.

On the initial examination, she appeared chronically ill but hemodynamic stable. Her vital signs were within normal limits. Cardiopulmonary and abdominal examinations were unremarkable. No signs of systematic infection were noted. She had a pain at upper cervical spine with a visual analog scale score (VAS) estimated of 4/10, aggravated by neck flexion. There was no evidence of torticollis sign. She was alert and oriented, with intact cranial nerves. Motor testing showed mild weakness in the upper extremities (grade 4/5) with preserved tone and bulk, and normal strength in the lower extremities, and there was no gait abnormality. Sensory examination revealed bilateral numbness involving both dorsal and palmar surfaces of hands with relatively preserved pain and temperature sensation. Increased deep tendon reflexes of biceps and triceps. Hoffmann’s sign was dynamically positive bilaterally. Clonus and Babinski’s sign were absent. Romberg’s test was negative.

Cervical CT-scan showed the subluxation of C2 odontoid process toward the center of the spinal canal at the level of C1–C2, indicating the spinal cord compression at current level. The patient has been definitively diagnosed with AAD causing spinal canal stenosis and spinal cord compression. Atlantoaxial screw fixation by Harms technique was initially planned. However, preoperative angiograph CT-scan revealed concurrent right vertebral artery hypoplasia (VAH) and left high-riding vertebral artery (HRVA). The anatomical variation of vertebral artery course posing a significant challenge to surgical approach and necessitated modification to the operative plan.

### Radiograph evaluation

**Figure 1.**
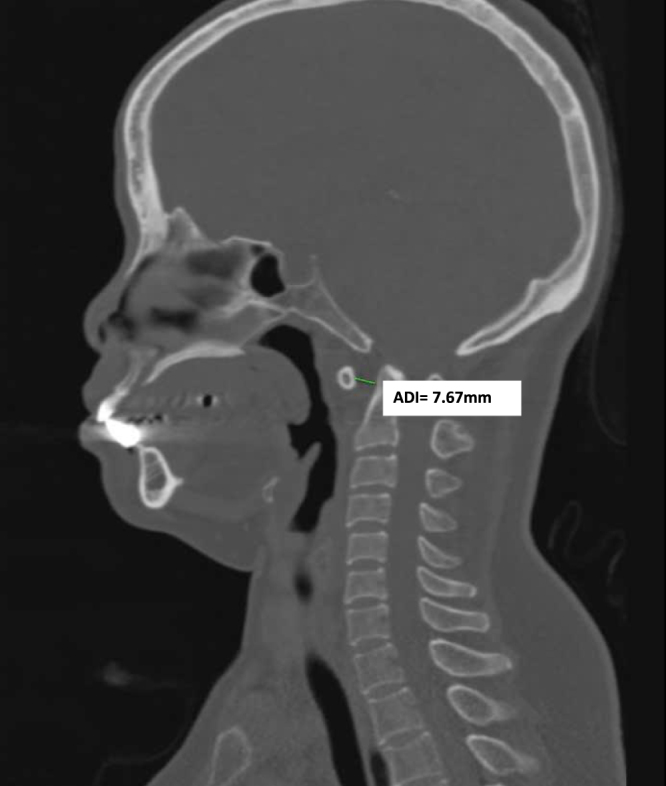
On CT-scan (bone window).

**Figure 2.**
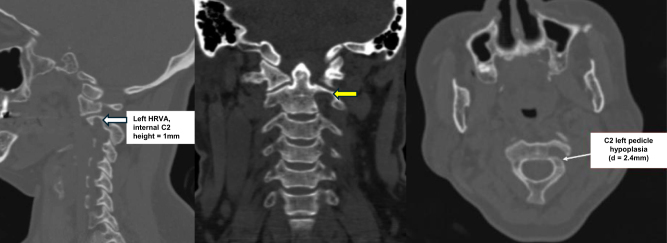
Cervical spine CT scan revealed the narrowing of the left C2 internal isthmus indicating left HRVA, and narrowing of C2 left pedicle.

**Figure 3.**
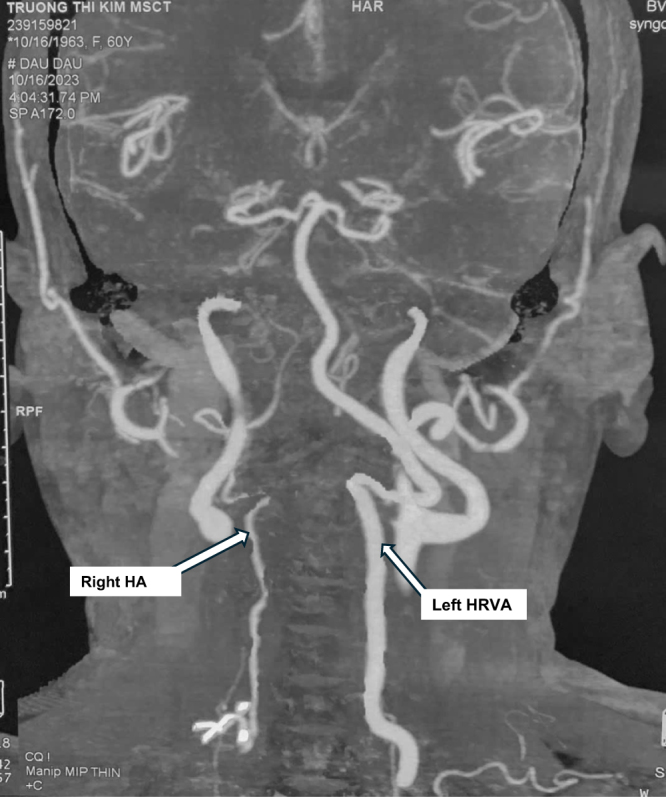
CT angiography demonstrated right hypoplasia of vertebral artery (HVA) with diameter of 1.5 mm and left HRVA.

**Figure 4.**
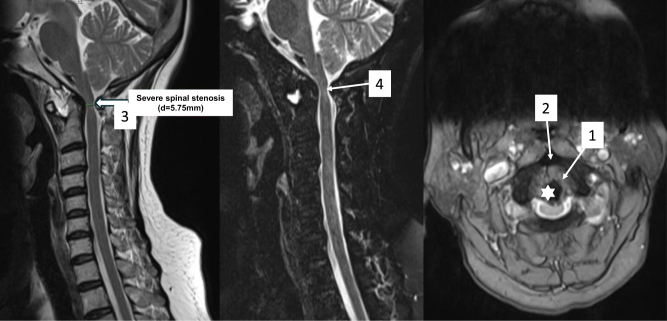
Cervical MRI revealed severe spinal stenosis at the level of C1–C2 (an anteroposterior diameter of 5.75 mm. On sagittal T2-STIR of MRI, the hyperintense signal within the spinal cord at the current level indicates spinal cord edema (arrow 4). On axial plane, the C2 odontoid process shifting to the center of the spinal canal directly compression to the spinal cord causing commensurate edema (star: C2 odontoid process; arrow 1: the interval between C1 anterior arch and C2 odontoid; arrow 2: C1 anterior arch; arrow 3: anteroposterior diameter of spinal cord).

CT-scan confirmed C1–C2 dislocation with ADI was 7.67 mm (Fig. 1), also demonstrated narrowing of the left C2 internal isthmus to 1 mm, indicating left HRVA, and a reduced width of the left C2 pedicle measuring 2.4 mm (Fig. 2). Preoperative CT angiography demonstrated a hypoplastic right vertebral artery (1.5 mm in diameter) and a normal size but high-riding left counterpart (HRVA), indicating increased risk for vertebral artery injury during posterior instrumental placement at the C1–C2 level (Fig. 3). Magnetic resonance imaging (MRI) illustrated spinal canal stenosis at the level of C2 odontoid process with anteroposterior diameter of spinal canal reduced to 5.75 mm (Fig. 4), which caused spine cord compression. No syrinx identified, however, there was narrowing of the subarachnoid space and intramedullary hyperintensity at the level of the cervical cord, consistent with spinal cord edema secondary to compression.

Cervical MRI revealed severe spinal stenosis at the level of C2 odontoid with an anteroposterior diameter of 5.75 mm. On sagittal T2-STIR of MRI, The hyperintense signal within the spinal cord at the current level indicates spinal edema (blue arrow). On axial plane, the C2 odontoid process shifting to the center of the spinal canal directly compression to the spinal cord causing commensurate edema (white star: C2 odontoid process, arrow 1: the interval between C1 anterior arch and C2 odontoid, arrow 2: C1 anterior arch, arrow 3: anteroposterior diameter of spinal cord; Fig. 4).

### Surgical treatment

Initially, the patient’s family was explained thoroughly about the risk of damage to left residual VA leading to compromise of cerebellar blood flow and cerebral ischemia resulting in death, and they consented to the procedure.

**Figure 5.**
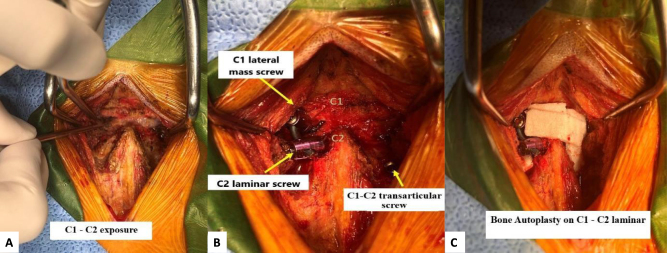
Intraoperative C1–C2 screw fixation steps. (A) C1–C2 exposure; (B) C1–C2 screw insertion technique; (C) an autologous iliac crest bone graft associated with artificial bone were placed on the posterior elements of C1 and C2.

In the operation theater, the patient was under general anesthesia in prone position with slightly extended and secured in a Mayfield frame. Cushion pads was positioned under the chest and iliac crests. The head positioning was adjusted under C-arm guidance to ensure the atlantodental interval (ADI) did not increase during the surgery. No intraoperative neuromonitoring (IONM) was available in our center; therefore, all steps were performed cautiously under continuous C-arm fluoroscopic guidance. Initially, posterior arch of C1 and the lamina of C2 were carefully exposed with dissection limited strictly to the posterior arch to avoid the region of the high-riding vertebral artery. Because of the left HRVA, trans-articular screw insertion as well as C2 pedicular screw placement posed a significant risk of vertebral artery injury. To minimize this risk, the surgical plan was modified to perform C2 laminar screw fixation (Wright’s technique) combined with C1 lateral mass screw. The entry point was located approximately 3 mm above the junction of C2 left laminar and C2 spinous process, centered on spinous process. Tapping was aligned parallel to the inclination of the right C2 lamina. The thickness of the C2 right laminar was measured as 5.03 mm on CT-scan. A 3.5 mm × 26 mm screw was inserted through the C2 laminar, and a 3.5 x 24 mm screw placed into C1 left lateral mass by Harms technique, as planned. On the right side, we performed C1–C2 trans-articular screw technique to reduce operative time. Finally, a high-speed burr was used to decorticate the posterior arch of C1 and posterior laminar of C2 bilateral side to prepare bone graft surface. An autologous iliac crest bone graft associated with artificial bone were placed on the posterior elements of C1 and C2 (Fig. 5A and B). Finally, a subfascial drain was inserted and the surgical incision was closed in anatomical layers, and a collar was applied postoperatively.

### Postoperative results

**Figure 6.**
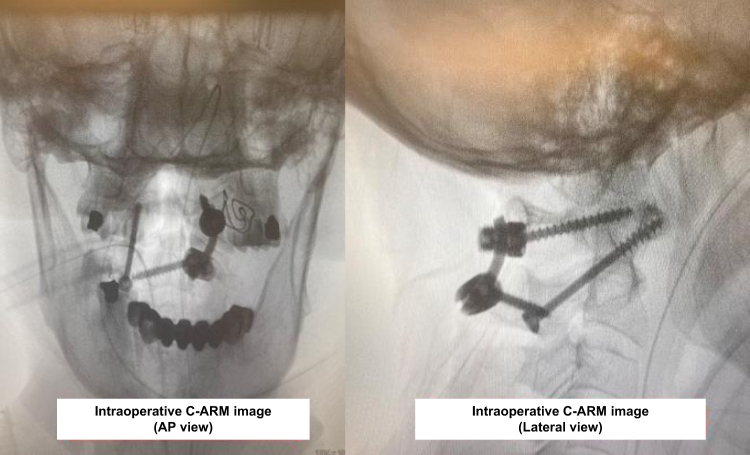
Intraoperative C1–C2 reduction under the guidance of C-arm.

**Figure 7.**
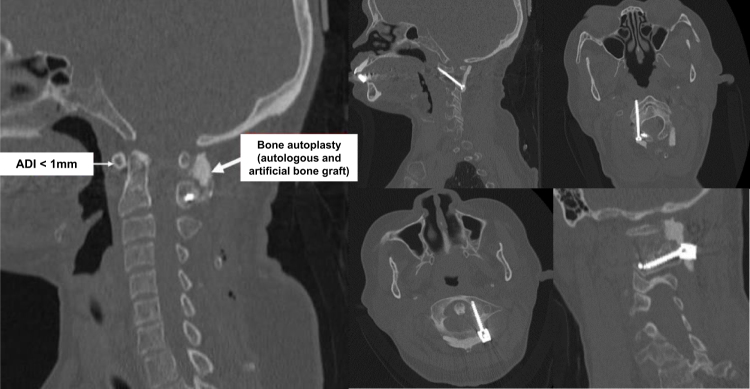
Postoperative CT scan revealed a good anatomical alignment reduction of C1–C2, with ADI reduced to 1 mm. All screws are inserted in the correct positions without penetrating the bone wall.

**Figure 8.**
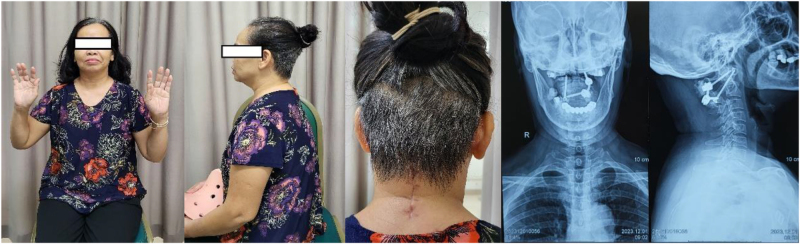
A 3-month follow-up examination showed a good initial outcome (no neurological deficit was found).

The total operation time was 95 minutes, and the total blood loss was 150 ml. The intraoperative C-arm images demonstrated good placements of screws (Fig. 6). The patient get out of bed on postoperative day 2, the patient’s symptoms improved remarkably with power in her muscles increasing to 5/5 on postoperative day 3, occipital region and upper extremity numbness declined gradually, and no progressive sphincter muscle dysfunction found. Postoperative CT scan showed the C1–C2 good anatomical alignment reduction (Fig. 7). The patient was discharged from hospital on postoperative day 7. After discharge, she was instructed to wear a cervical collar intermittenly for a duration of 4 weeks to support postoperative stabilization. At 3-month follow-up visit, she had sustained good clinical outcome, with full neurological recovery and no recurrent symptoms (Fig. 8).

## Discussion

The stability of the C1–C2 complex depends not only on osseous but also on the ligamentous structure^[^2^]^. For nontraumatic causes, there must be some degree of ligamentous damage along with bone lesions, therefore closed reduction by Halo traction followed by surgical fixation of C1–C2 is recommended to ensure the stability of the atlantoaxial complex^[^8,9^]^. In case of nontraumatic AAD, a definite diagnosis could be difficult because of non-specificity of symptoms, and it often diagnosed incidentally in the course of investigating patient with worsening neurology almost case. For our patient, she has received treatment in Rheumatology Center for several times, and all rheumatoid markers are negative. Unfortunately, we do not have any evidence of gene disorder confirmed on the patient. On this case, a meticulous clinical examination revealed the reduced fine motor function of the upper extremities, and the positive dynamic Hoffman sign on both sides suggested a subtle cervical cord compression. Although the Hoffman sign could be positive at the proportion of 0.3–2% in normal individuals^[^10,11^]^, the dynamic Hoffman sign accompanied by sensorimotor deficit is a useful sign aiding physicians to suspect and detect early spinal cord compression^[^12^]^.

There are many options for C1–C2 surgical screw fixation, for instance, the Harms technique, Magerl’s transarticular screw (TAS) technique^[^1], or even hybrid constructs selected for this case.

In terms of vertebral artery variations, AVH prevalence is reported to vary between 1.9 and 26.5% in the literature and is incidentally detected in asymptomatic healthy people. According to Morimoto^[^13^]^, the rate of unilateral distal segment VAH was reported at 0.2%, after combining Basiparallel Anatomical Scanning MRI and MRA in the asymptomatic population, the figure for distal vertebral artery hypoplasia is 4.6%. HAV is defined by a VA diameter of ≤2 mm, and more frequent on the right side^[^14^]^. Besides, it is considered a risk factor for circulation ischemia, especially in the area supplied blood by the posterior inferior cerebellar artery^[^14^]^. For HRVA, the global prevalence of this artery course anomaly is estimated at 20.9%^[^15^]^, and this rate doubles in patients with rheumatic arthritis^[^16^]^. HRVA is diagnosed when the C2 isthmus diameter is ≤5 mm and/or the internal height of the pedicle within the C2 vertebra is ≤2 mm^[^17^]^. According to Yeom^[^18^]^, half of the cases with HRVA would have a narrowing of the C2 pedicle, while most cases of C2 narrowing pedicle could go along with HRVA. Maki *et al*^[^19^]^, suggest that the screw fixation technique via C2 pedicle is not feasible when the diameter of the pedicle is equal to or less than 4 mm. Marques *et al*^[^20^]^, utilizing multiplanar reconstruction with the OsiriX system, determined that to insert a 3.5-mm screw through a C2 pedicle, the minimum diameter of the pedicle should be at least 5.5 mm^[^21^]^, and a different experiment using 3D printing reconstruction technology showed the likelihood of this figure should be at least 4.78 mm.

The overall rate of iatrogenic vertebral artery injury varies from 0.08 to 1.4% in cervical spine surgery, more frequent in the posterior approach to C1–C2 (4.1–8.2%)^[^22^]^. In our patient, the diameter of the left C2 isthmus and VA pedicle are 2 mm and 2.8 mm, respectively. For that reason, TAS or pedicle screw was a risky option. One documented case in the literature involved AAD along with right AVH, the proposed treatment was transoral decompression and atlantoaxial posterior fusion. Unfortunately, this intervention was unsuccessful, and the patient passed away 5 days after the surgery due to brain edema followed by a compromise of intracranial circulation^[^5^]^. Even though VA injury followed the TAS technique quite highly (8.2%)^[^23^]^, some authors reported successful C1–C2 TAS placement after artery loop mobilization^[^24^]^. HRVA is a contraindication to the C1–C2 TAS^[^25^]^.

The incidence of VA injury, in case of HRVA, due to C2 pedicle screw is 5.3–21%^[^18^]^, making the option of C2 pedicle screw fixation on the left side inappropriate in this case. To solve the issue, we opted to perform a hybrid technique with C1 lateral mass screw placement combined with C2 translaminar screw technique (Wright technique) to avoid placing the residual VA at risk, and C1–C2 TAS on the right side to reduce operation time. Some studies indicate that screw insertion through the pedicle and laminar offered similar biomechanical properties, providing stability to the atlantoaxial complex while minimizing the risk of VA injury^[^23,26,27^]^. Another research proposal implies that surgeons have the flexibility to choose either approach and can expect similar anatomical risks of vertebral artery injury with anterior percutaneous TAS fixation^[^28^]^. However, our department has the expertise for treating severe AAD via the posterior approach; hence, we chose the mentioned method to optimize the successful rate of this operation.

This report emphasizes the technical challenge of instrumentation in the setting of a single dominant vertebral artery, where inadvertent injury can be catastrophic. Careful identification of vertebral artery anatomy using CT-angiography and the use of hybrid fixation techniques are essential to avoid complications. Even in the absence of intraoperative neuromonitoring (IONM), adherence to strict anatomical dissection and fluoroscopic control can help achieve a safe outcome.

## Conclusion

Atlantoaxial fixation in non-traumatic atlantoaxial dislocation with high-riding and hypoplastic vertebral artery carries a high risk of vascular injury. Comprehensive vascular assessment and individualized planning are essential to achieving safe instrumentation.

## Data Availability

Data supporting the findings of this study are available within the article. No additional datasets were generated or analyzed.
